# Perceived must see pediatric conditions vs. actual clinical exposure during an undergraduate pediatrics clerkship

**DOI:** 10.3389/fmed.2026.1876315

**Published:** 2026-10-07

**Authors:** Hamed Al Ahbabi, Omar Tafech, Hassan Ballas, Mohammed Al-Zaabi, Mohammed Al-Blooshi, Ahmed Alsuwaidi, Elhadi H. Aburawi, S. Ayhan Çalışkan, Awad Al Essa, Imran Zafar, Sami Shaban, Mohi Eldin Magzoub

**Affiliations:** College of Medicine and Health Sciences, United Arab Emirates University, AlAin, United Arab Emirates

**Keywords:** clinical exposure, COMSEP, curriculum reform, logbooks, pediatric clerkship, required clinical experiences

## Abstract

**Background:**

Undergraduate pediatric rotations aim to provide authentic exposure to core conditions. Programs often publish “must-see” lists, but student and doctor perceived priorities may not reflect real-world case-mix. We compared student/doctor perceived must-see topics with the diagnoses and presentations they actually encountered.

**Methods:**

Cross-sectional comparison of a perception dataset (49 participants: 39 medical students and 10 doctors; 81 presentation-illness pairs, mean ± SEM on a 1–5 scale) and an electronic logbook of clinical encounters. Free-text logbook fields were normalized, segmented, and classified as presentations or diagnoses by the authors and were not independently reviewed. We computed documented diagnosis and presentation frequencies, mapped survey pairs to observed labels, and examined alignment using Spearman's rank correlation coefficient. Mapping coverage and discrepant priority-exposure patterns were summarized descriptively.

**Results:**

The logbook comprised 5,179 encounters. After normalization and mapping, 63% (51/81) of survey pairs had at least one documented match. Spearman's rank correlations were *ρ* = 0.713 for diagnosis pairs (*n* = 15, *p* = 0.003, 95% CI = 0.32–0.90) and *ρ* = 0.087 for presentation pairs (*n* = 11, *p* = 0.800, 95% CI = −0.54–0.65). Respiratory and gastrointestinal conditions were prominent in both datasets. No mapped logbook entry was found for innocent/pathologic heart murmur, but this may reflect limited exposure, documentation omission, or mapping/capture error rather than confirmed absence. Hydronephrosis was documented 12 times despite a perceived rank of 70.

**Conclusions:**

The logbook documented frequent exposure to several common core conditions that students and doctors valued, while some high-priority items had few or no mapped entries. Because absence from a free-text logbook is not proof of no clinical exposure, structured chief-complaint and final-diagnosis fields, mapping validation, targeted simulation/virtual cases, and data-informed scheduling may improve both documentation and equitable curricular coverage.

## Introduction

Clinical rotations are a cornerstone of undergraduate pediatric education, providing authentic exposure to conditions and presentations that build diagnostic reasoning and patient-care skills. Programs often define expectations through “must-see” lists, but actual exposure varies with case-mix, seasonality, and clinical site. To align priorities with real-world experience, perception must be examined alongside observed encounters. Furthermore, accreditation standards require medical schools to define, and monitor required clinical experiences and to ensure equitable access to alternatives when clinical opportunities are limited (1–3).

In pediatrics, the Council on Medical Student Education in Pediatrics (COMSEP) curriculum articulates core presentations and illnesses and is widely used to anchor clerkship objectives and assessment. Programs frequently supplement variable clinical exposure with vetted cases such as COMSEP Clinical Cases and Aquifer Pediatrics, which map directly to COMSEP objectives and standardize coverage across seasons and sites (4–6).

Encounter logbook often used to document exposure are a form of mandated self-report and show weak or inconsistent associations with downstream performance. Studies in internal medicine and surgery clerkships report minimal correlation between log volume and Objective Structured Clinical Examination (OSCE)/National Board of Medical Examiners (NBME) exams or final grades, and implementation research emphasizes that logs improve learning only when linked to clear use cases, feedback, and engaged supervision (7–11).

Observed exposure is shaped by case-mix and seasonality. Bronchiolitis, for example, shows winter-predominant incidence and pandemic-era shifts in timing and volume across children's hospitals. Authoritative reviews and guidelines also note that evidence-based pediatric care can emphasize supportive management, limiting opportunities to witness procedures (12–15).

To close predictable exposure gaps, programs increasingly deploy virtual patients and technology-enhanced simulation. Systematic reviews and meta-analyses demonstrate improved knowledge, clinical reasoning, and skills compared with traditional instruction or no intervention; in pediatrics, a randomized trial shows that a brief multimedia auscultation module improves recognition of pediatric heart murmurs (16–20).

Accordingly, we compared student/doctor perceived must-see list with conditions and presentations documented in pediatric-rotation logbooks. Our objectives were to: (i) describe perceived importance across common pediatric presentation-illness pairs; (ii) quantify documented diagnosis and presentation frequencies; (iii) estimate the association of perceived importance with diagnosis frequency and, separately, presentation frequency; (iv) compare the strength and interpretability of those two associations; and (v) identify targeted opportunities for curricular refinement. These analyses were exploratory; no directional hypothesis was preregistered.

## Methods

We conducted a cross-sectional analysis comparing (1) student and doctor perceptions of must-see topics from a structured survey of 49 participants [39 medical students and 10 doctors; 81 presentation-illness pairs (21), reported as mean ± SEM on a 1–5 scale] and (2) clinical exposure documented in students' logbooks. in which they have independently observed clinical exposure. Participants were recruited during the 2024/2025 academic year. The students were in the fifth year of a six-year undergraduate MD program and had completed a pediatric rotation.

A presentation was defined as a symptom, sign, or chief-complaint label, whereas a diagnosis was a named disease or condition documented as an assessment or final diagnosis. Free-text fields likely to contain either category were concatenated per encounter, converted to lowercase, Unicode and punctuation variants were normalized, and abbreviations were expanded. Then, matching was done by the authors using the normalized presentation and diagnosis from the logbook exactly matched with the must-see list. Composite survey presentations (e.g., “cough and wheeze”; “fever and rash”) were treated as two presentations for comparison. A mapped count represents a documented label occurrence, not necessarily a unique encounter or independently verified clinical event.

The primary outcome was Spearman's rank correlation coefficient between mean perceived-importance rating and documented diagnosis frequency across survey presentation-illness pairs. Secondary outcomes were the corresponding correlation with presentation frequency, mapping coverage, high-perceived but rarely documented items, and lower-perceived but frequently documented items. Average ranks were assigned to ties, and two-sided *p* values were calculated. 95% confidence intervals were calculated using Fisher's z transformation. “NA” in Tables 1, 2 denotes a documented label without a corresponding item in the 81-pair must-see list cross-reference; such labels were retained descriptively but had no perceived rank and were excluded from rank-alignment calculations. Counts are mapped label occurrences and are expressed per 100 encounter rows. The perceived ranks came from the combined student/doctor ratings.

**Table 1:** Top 20 observed clinical presentations as documented in the student logbooks (*n* = 970).

Diagnosis	Count	Percent	Observed Frequency Rank	Perceived must-see rank (student/doctor survey)	Difference (must-see rank minus observed rank)
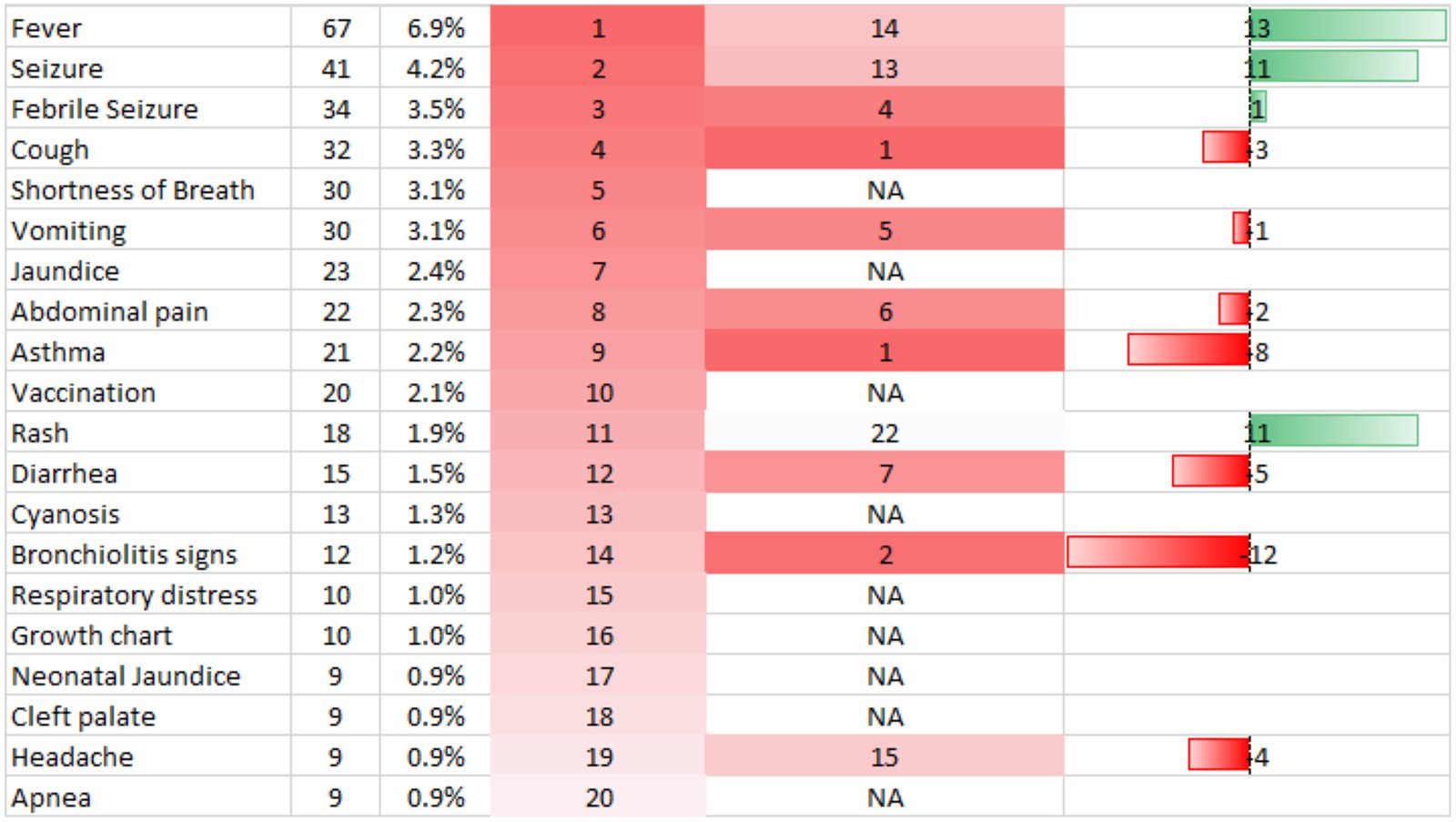					

**Table 2:** Top 22 observed diagnoses as documented in the student logbooks (*n* = 4,335)

Presentation	Count	Percent	Observed Frequency Rank	Perceived must-see rank (student/doctor survey)	Difference (must-see rank minus observed rank)
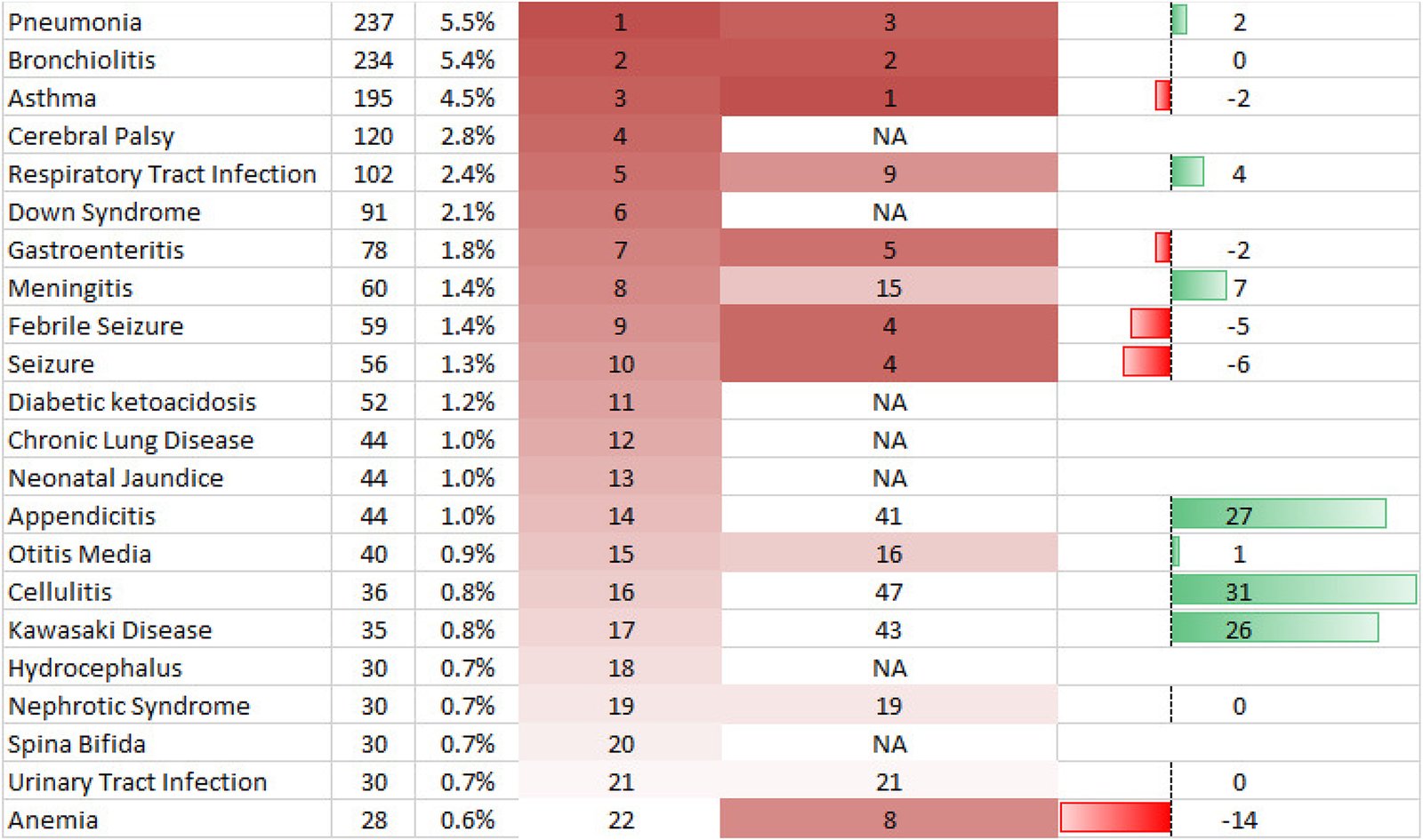					

### Ethics

This human-participant study was approved by the United Arab Emirates University Institutional Review Board [ERSC_2025_8089, 5/8/2025]. Completion of the online survey indicated informed consent. No identifiable personal data were collected.

## Results

The logbook contained 5,179 encounter rows. Mapping produced 970 presentation occurrences and 4,335 diagnosis occurrences; these denominators are mapped occurrences rather than unique encounters, and one encounter could contribute more than one segment or label. The perception dataset comprised 81 presentation-illness pairs with combined student/doctor ratings. Because we normalized the logbook entries, we were able to directly match all logbook entries with the must-see list. Those entries in the logbook that are not in the must-see list were checked by the authors and verified not to match any of the must-see list items. No independent review of this normalization or matching was deemed necessary as this fell within the authors' expertise and was a straightforward task.

Tables 1, 2 summarize the most frequently mapped presentation and diagnosis labels, respectively. Table 3 identifies high-perceived items with few or no mapped entries, and Table 4 identifies lower-perceived items with more than 10 mapped diagnosis occurrences. Because these tables combine survey items with labels found only in the logbook, unmatched labels are displayed as NA. Such entries remain descriptive exposure observations but are excluded from rank-paired correlation calculations. We have compared the perceived combined student/doctor must-see list with the student logbook entries ranking.

**Table 3:** High-perceived items with few or no mapped logbook entries (top-22 perceived rank; 0 or 1 mapped count).

Presentation	Diagnosis	Must-See Perceived Rank (out of 81)	Observed Presentation/Diagnosis
Heart murmur	Innocent and pathologic murmur	10	0/0
Proteinuria	Nephrotic syndrome	19	0/30
Otalgia	Otitis media	16	1/1

**Table 4:** Low-perceived (Ranked in bottom 22 must-see perceived list) but commonly seen (>10 observations)

Diagnosis	Observed Diagnosis Count	Must-See Perceived Rank (out of 81)
Hydronephrosis	12	70

The top 20 presentation labels accounted for 434 of 970 mapped presentation occurrences (44.7%). Nine labels had no counterpart in the 81-pair survey and therefore had no perceived rank. All 11 matchable labels were ranked within the survey's top 22.

The top 22 diagnosis labels accounted for 1,675 of 4,335 mapped diagnosis occurrences (38.6%). Seven labels had no counterpart in the 81-pair survey. Of the 15 matchable labels, 12 were ranked within the survey's top 22 (80.0% of the matchable subset); appendicitis (rank 41), Kawasaki disease (rank 43), and cellulitis (rank 47) were outside the top 22. Unmatched labels were excluded from this rank comparison.

Spearman's rank-order correlation showed a strong, statistically significant positive association between the observed frequency and perceived must-see ranks for diagnoses (*ρ* = 0.713, *n* = 15, *p* = 0.003, 95% CI = 0.32–0.90). In contrast, there was no significant association between the observed frequency and perceived must-see ranks for clinical presentations (*ρ* = 0.087, *n* = 11, *p* = 0.800, 95% CI = −0.54–0.65). Cases containing NA values were excluded pairwise and average ranks were assigned to tied values.

## Discussion

We compared student/doctor perceived must-see pediatric topics with student exposure documented in electronic logbooks. Respiratory and gastrointestinal conditions were prominent in both datasets. The diagnosis-level correlation was positive, but its confidence interval was wide; the presentation-level interval included moderate negative and positive associations. The apparent difference between the two coefficients was not formally tested and should not be interpreted as evidence that one level aligns better than the other. Seasonal explanations, including winter-predominant bronchiolitis, remain plausible but cannot be tested without date- and season-stratified data (12–15).

Our results extend prior work on required encounters and logbooks. Earlier studies reported weak or absent associations between log volumes and downstream clerkship performance or exam scores, highlighting the limits of mandated self-report as a proxy for competence (7–9). Implementation research further cautions that simply introducing a logbook, without clear use cases, feedback, and engaged precepting, may not improve learning and can even lower perceived supervision quality (10, 11). In contrast, our focus was not on predicting performance from volumes but on aligning perceived importance with observed case mix, which can more directly inform curricular design.

A recurring theme across programs is documentation quality. Free-text entries can blur presentations with diagnoses and invite abbreviations, misspellings, negation, and incomplete physical-examination documentation. Accordingly, an absent mapped term cannot distinguish non-exposure from non-documentation or mapping failure. The findings therefore support structured dual-entry (chief complaint and final diagnosis) with controlled vocabularies and synonym pick-lists, consistent with implementation guidance and accreditation expectations to define, monitor, and remediate required clinical experiences (1–3, 11).

Potential exposure gaps may be appropriate targets for engineered learning, but they must first be distinguished from documentation and mapping gaps. In particular, the lack of a mapped murmur entry should not be interpreted as confirmed absence unless the logbook fields were designed to capture physical-examination findings and a record audit supports that interpretation. Evidence indicates that virtual patients and technology-enhanced simulation improve knowledge, clinical reasoning, and skills across health professions education (16–19). In pediatrics, a randomized trial showed that a short multimedia auscultation module improves medical students' recognition of pediatric heart murmurs (20). Once gaps are verified, these approaches and COMSEP-aligned virtual cases may standardize coverage across seasons and sites (4–6).

This study has several limitations. The perception sample was small (49 participants: 39 medical students and 10 doctors) and drawn from one institution; the logbooks covered inpatient encounters across multiple training sites. Survey ratings reflect perceived importance rather than competence, and logbooks are self-reported documentation rather than independently verified exposure. The mapping process was not validated against an independently coded reference sample. The correlation analyses also had small effective paired samples (*n* = 15 and *n* = 11), producing wide confidence intervals. Accordingly, the findings are exploratory and should not be used to infer curricular adequacy or causal relationships. Future work should use multicenter data, structured electronic logbooks, transparent lexicons and algorithms, and prospectively validated mapping (10, 11).

## Implications for curricular reform of pediatric rotations

- Guarantee exposure to low-incidence, high-importance conditions via simulation, standardized patients, and brief case-based modules (e.g., cardiac auscultation for murmurs; edema/proteinuria work-ups for nephrotic syndrome).
- Adopt structured logbook entries with dual fields, chief complaint/presentation and final diagnosis, backed by controlled vocabularies and synonym/abbreviation pick-lists; include flags for uncertainty and negation.
- Provide learner dashboards visualizing exposure against the must-see list (traffic-light coverage, site breakdowns) to trigger targeted activities.
- Balance placements across sites and seasons to broaden case-mix; where narrow, add micro-rotations or focused half-day experiences.
- Integrate skills labs aligned with exposure gaps (e.g., auscultation, dermatology pattern recognition, urine dip interpretation) followed by OSCEs.
- Close the loop with faculty via pocket guides that translate the must-see list into teaching prompts and checklists.
- Institutionalize annual data review to update the must-see list and logbook vocabulary based on observed trends and stakeholder feedback.

## Conclusion

The logbook documented frequent exposure to several common core conditions that students valued, while some high-priority items had few or no mapped entries. Because absence from a free-text logbook is not proof of no clinical exposure, structured chief-complaint and final-diagnosis fields, mapping validation, targeted simulation/virtual cases, and data-informed scheduling may improve both documentation and equitable curricular coverage. By adding structured documentation, validated mapping, targeted simulation, and data-informed scheduling, programs can identify and address verified gaps while avoiding overinterpretation of missing free-text entries.

## Data Availability

The raw data supporting the conclusions of this article will be made available by the authors, without undue reservation.
